# Neoadjuvant Chemotherapy and Targeted Therapy in Breast Cancer: Past, Present, and Future

**DOI:** 10.1155/2013/732047

**Published:** 2013-08-20

**Authors:** Simon P. Gampenrieder, Gabriel Rinnerthaler, Richard Greil

**Affiliations:** 3rd Medical Department with Hematology, Medical Oncology, Hemostaseology, Rheumatology and Infectious Diseases, Oncologic Center, Laboratory of Immunological and Molecular Cancer Research, Paracelsus Medical University, Müllner Hauptstraße 48, 5020 Salzburg, Austria

## Abstract

Traditionally, neoadjuvant treatment for breast cancer was preserved for locally advanced and inflammatory disease, converting an inoperable to a surgical resectable cancer. In recent years, neoadjuvant therapy has become an accepted treatment option also for lower tumor stages in order to increase the rate of breast conserving therapy and to reduce the extent of surgery. Furthermore, treatment response can be monitored, and therefore, patient compliance may be increased. Neoadjuvant trials, additionally, offer the opportunity to evaluate new treatment options in a faster way and with fewer patients than large adjuvant trials. Compared to the metastatic setting, the issue of acquired resistance and pretreatments, which may distort treatment efficacy, can be avoided. New trial designs like *window-of-opportunity* trials or *postneoadjuvant* trials provide the chance to identify tumor sensitivity or to overcome tumor resistance in early tumor stages. In particular, in HER2-positive breast cancer, the neoadjuvant approach yielded great successes. The dual HER2 blockade with trastuzumab and pertuzumab recently showed the highest pCR rates ever reported. Many new drugs are in clinical testing with the aim to further increase pCR rates. Whether this endpoint really represents a surrogate for long-term outcome is not answered yet and will be discussed in this review.

## 1. Introduction

In recent years, there has been a growing interest in the neoadjuvant approach for early breast cancer. Compared to the classical adjuvant treatment, it offers several advantages. First, it provides the opportunity to monitor response during treatment and allows changing or discontinuing treatment in case of nonresponsiveness. Even if an advantage by changing therapy has not yet been proven, toxicity of an ineffective treatment can be avoided. The demonstration of treatment efficacy, conversely, motivates patients to continue therapy despite toxicities. Second, the rate of breast conservation can be increased, and in case of breast conserving therapy (BCT), the extent of surgery can be reduced. Additionally, primarily inoperable tumors can be downsized allowing a curative intervention. Third, the residual cancer burden (RCB) is a powerful prognostic marker, sometimes changing the initial prognostic profile in either way. Forth, in neoadjuvant trials predictive markers, tumor biology, mechanisms of resistance, and new treatment approaches can be investigated more rapidly and with fewer patients than in adjuvant studies.

In the following sections, we give an overview of the historical background which is the basis for current treatment and research strategies. We discuss context and data of recent trials and give a perspective to future developments.

## 2. Neoadjuvant versus Adjuvant

In the early nineties, neoadjuvant and adjuvant therapies were first compared in randomized trials, using the same chemotherapeutic regimen pre- and postoperatively. The initial intention was to improve long-term outcome in patients with large but potentially operable breast cancer due to an earlier exposure to systemic therapy. The largest and most important trial was the *National Surgical Adjuvant Breast and Bowel Project (NSABP) B-18* trial which compared 4 cycles of doxorubicin plus cyclophosphamide (AC) given either preoperatively or postoperatively. In total, 1,523 women with a median tumor size of 3.5 cm were included independent of hormone receptor status. In the neoadjuvant arm, the objective clinical response (ORR) rate was 78% with clinical partial response (cPR) in 43% and a clinical complete response (cCR) in 36%. A pathologic complete response (pCR) was documented in 13% of patients. The two main findings in *NSABP B-18* however were (1) no difference in overall survival (HR = 0.99; 95% CI, 0.85 to 1.16; *P* = .90) and disease-free survival (HR = 0.93; 95% CI, 0.81 to 1.06; *P* = .27) between pre- and postoperative chemotherapy; (2) patients achieving a pCR had a superior DFS and OS compared to patients not achieving a pCR (DFS: HR = 0.47, *P* < .0001; OS: HR = 0.32, *P* < .0001). Furthermore, there was a trend in favor of neoadjuvant chemotherapy compared with adjuvant therapy for OS and DFS in women younger than 50 years (OS: HR = 0.81, *P* = .06; DFS: HR = 0.85, *P* = .09) [1]. 

Similar results were shown in the *EORTC trial 10902* where 698 patients (T1c-T4b, N0-1, M0, ER positive and negative) were randomly assigned to 4 cycles of fluorouracil, epirubicin, and cyclophosphamide (FEC) administered preoperatively versus the same regimen given postoperatively. There was no difference in terms of PFS, OS, or local recurrence rate (HR 1.15, *P* = .27; HR 1.16, *P* = .38; and HR 1.13, *P* = .61, resp.) [2]. Nine randomized studies comparing neoadjuvant with adjuvant treatment were pooled in a meta-analysis including a total of 3,946 patients. Similar to the previously mentioned phase-III trials, Mauri and colleagues found no difference with regard to death (RR 1.00, 95% CI, 0.90 to 1.12), disease progression (RR 0.99, 95% CI, 0.91 to 1.07), or distant disease recurrence (RR 0.94, 95% CI, 0.83 to 1.06). However, the rate of local recurrence was higher in the neoadjuvant group (RR 1.22, 95% CI, 1.04 to 1.43). This was mainly caused by trials in which surgery was avoided in case of clinical complete response [3].

Nonetheless, for an individual patient, the delay of surgery by preoperative therapy could provide potential harm. Given that all randomized trials are comparisons of cohorts, the disadvantages of single patients are not reflected in the overall results. Fortunately, the proportion of tumors progressing during neoadjuvant therapy is very low, but hypothetically even if the tumor as a whole is shrinking, single tumor cells could respond differentially. As discussed elsewhere, partly resistant tumor cells might acquire full-blown resistance during neoadjuvant treatment and generate micrometastases [4].

In summary, the primary objective to show an advantage due to earlier systemic therapy was not met, but it has been shown that neoadjuvant chemotherapy is as effective as adjuvant chemotherapy. Additionally, the rate of breast conservation in operable disease can be increased, even if the risk of local recurrence might be slightly higher. 

The results of trials comparing neoadjuvant with adjuvant chemotherapy are summarized in Table 1.

## 3. Addition of Taxanes

The rate of pCR in these early trials was quite low with a range from 4 to 29%. Therefore, the addition of taxanes to the classical anthracycline-based chemotherapy was investigated in several phase-III trials. In 2002, first results from the *Aberdeen* trial (*n* = 162) were reported, where 4 cycles of cyclophosphamide, doxorubicin, vincristine and prednisolone (CVAP) followed by four cycles of docetaxel were compared with eight cycles of CVAP. All clinical endpoints were better in the taxane containing arm, including a higher rate of clinical response (66 versus 94%, *P* = .001), pCR (15 versus 31%, *P* = .06), an improved 5-year OS (78 versus 93%, *P* = .04) and 5-year DFS (72 versus 90%, *P* = .04), and a higher rate of BCT (49 versus 67%) [5]. In contrast, in the large *NSABP B-27* trial, the addition of docetaxel showed no difference in DFS, OS, and rate of BCT. Here, a total of 2,411 patients were randomized between 4 cycles of doxorubicin/cyclophosphamide (arm 1), the same schedule followed by 4 cycles of docetaxel preoperatively (arm 2) or postoperatively (arm 3). Patients in arm 2 had a higher clinical (64%) and pathological complete response rate (26%) than patients in arms 1 and 3 (40% and 14%, resp.). Additionally, a trend towards longer relapse-free survival (RFS) was observed for arm 2 in comparison with arm 1 (5-year RFS: 74 versus 70%, HR 0.85; 95% CI, 0.71 to 1.02). Again, pCR was a highly significant surrogate marker for better outcome [1]. Seven randomized trials including 2,455 patients were summarized in a literature-based meta-analysis in order to answer the question if the addition of taxanes to an anthracyclines-based chemotherapy provides an advantage in the primary treatment for early breast cancer. The rate of BCT was significantly higher for patients receiving taxanes, with an absolute difference (AD) of 3.4% (*P* = .012). The rate of pCR was higher for patients receiving taxanes, but only statistically significant if used in a sequential schedule with an AD of 2.4% (*P* = .013) [6].

The results of randomized trials incorporating either concurrent or sequential taxane-based neoadjuvant therapy are summarized in Table 2.

## 4. Addition of Other Chemotherapeutics

Beside taxanes, several other chemotherapeutics were added to neoadjuvant regimens to further improve pCR rate. In the *TOPIC* trial, the efficacy of continuous infusional 5-fluorouracil for 18 weeks and the integration of cisplatin were investigated. In comparison to 6 cycles of doxorubicin plus cyclophosphamide, no difference in response rate (75 versus 77%, *P* = .6), pCR rate (16 versus 16%, *P* = 1.0), or long-term outcome (5-year OS 82 versus 74%, *P* = .18) could thereby be shown. Infusional 5-FU, however, was associated with a higher rate of grade 3 toxicities [7]. Similar results were shown in the *TOPIC-II* trial investigating epirubicin plus vincristine versus standard AC without improving any of the endpoints (cORR, pCR, BCT, DFS, and OS) [8]. Negative results were also found for gemcitabine investigated in the *Neo-tAnGo* and the *NSABP B-40* trials [9, 10]. For capecitabine, available data are contradictory: in the ABCSG-24 study, the addition of capecitabine to 6 cycles of epirubicin plus docetaxel improved the pCR rate (24 versus 16%, *P* = .02) [11], while in the *NSABP B-40* and *GeparQuattro* trials no differences in pCR rates were observed. In *NSABP B-40*, capecitabine was administered for 3 cycles in addition to docetaxel followed by AC [10] and in *GeparQuattro* capecitabine was given in combination or sequentially to docetaxel after 4 cycles of AC [12]. The longer administration of capecitabine (6 versus 4 cycles) and the missing alkylating agent in ABCSG-24 are two potential explanations for these differences. Based on these results, neither capecitabine nor 5-FU, cisplatin, vinorelbine, or gemcitabine can be considered as standard neoadjuvant therapy in operable breast cancer.

## 5. PCR as a Prognostic Marker and as a Surrogate for Long-Term Outcome

In many neoadjuvant trials, patients achieving a pCR showed a better long-term outcome, indicating pCR as a strong prognostic marker [1, 13]. This is particularly true if both breast and lymph nodes are free of invasive carcinoma. The influence of residual intraductal disease (DCIS) on prognosis is not yet totally clear. In a retrospective analysis of 2,302 breast cancer patients treated in a neoadjuvant setting at the MD Anderson, 3.4% had pCR in breast and lymph nodes and 8.6% had residual DCIS. There was no difference in terms of 10-year DFS rates (81 versus 82%), 10-year OS rates (92 versus 93%), and locoregional RFS rates (93 versus 91%) [14]. In contrast, a pooled analysis of 7 randomized trials conducted by the German Breast Group (GBG) including 6,377 patients showed a small but significant difference in DFS between patients without any residual disease (ypT0/N0) and patients with remaining DCIS (ypTis ypN0) (HR 1.74; 95% CI, 1.28 to 2.36; *P* = .001). In addition, a trend towards better OS was shown (HR 1.41; 95% CI, 0.87 to 2.29; *P* = .166) [15]. The same analysis demonstrated that in luminal A and luminal B (ER plus HER2 positive) tumors, pCR is not associated with prognosis, whereas in patients with highly proliferating carcinomas like triple negative breast cancer (TNBC) or HER2 enriched tumors (HER2 positive plus ER negative), pCR can accurately discriminate between good and poor prognosis [15].

The prognostic value of pCR for an individual patient has to be distinguished from the value of pCR as a surrogate for long-term outcome in neoadjuvant trials. This precondition is however crucial, because pCR is the primary endpoint of almost all neoadjuvant trials. At best, an advantage in pCR rate should translate into better DFS and OS. This has been perfectly proven in the *NOAH* trial where the addition of trastuzumab not only showed a higher pCR rate in the breast (ypTis/Nx: 43 versus 22%, *P* = .0007) but also a significantly higher 3-year event-free survival (EFS) (71 versus 56%, *P* = .013) [16]. In contrast, the addition of docetaxel to AC in *NSABP B-27* led to higher pCR (ypTis/N0) rates but did not influence DFS or OS [1]. The lack of correlation might be explained by the inclusion of low proliferative subtypes where pCR is not only rare but even unassociated with survival.

The question whether pCR really displays a reliable surrogate endpoint to replace survival data cannot be finally answered yet. The FDA has now initiated a pooled analysis of over 12,000 patients treated in different clinical trials to answer this question and to allow accelerated approval procedures for therapeutics showing a significant improvement in pCR rate in high risk operable breast cancer.

## 6. Response-Adapted Therapy

In contrast to adjuvant therapy, preoperative treatment allows to monitor response in breast and lymph nodes and gives the opportunity to discontinue or change treatment in case of nonresponsiveness. This approach was investigated in two large phase-III trials by the GBG. Both trials used ultrasound to assess treatment response. This is crucial, because clinical assessment by palpation frequently overestimates treatment response [17].

The *GeparTrio* trial evaluated the impact of adapting therapy after 2 cycles of docetaxel/doxorubicin/cyclophosphamide (TAC) depending on response. In case of nonresponsiveness, defined as sonographic reduction in the product of the two largest perpendicular diameters by less than 50%, patients were randomly assigned to receive either further 4 cycles of TAC (standard arm) or 4 cycles of vinorelbine/capecitabine (NX, response guided arm). Responders were randomized between further 4 (standard arm) and further 6 cycles of TAC (response guided arm), respectively. Interestingly, response-adapted therapy did not change pCR rate (6x versus 8x TAC: 21 versus 24%, *P* = .27; 6x TAC versus TAC-NX: 5 versus 6%, *P* = .73); however, it prolonged DFS and OS significantly (6x TAC versus 8x TAC/TAC-NX: HR 0.71, 95% CI, 0.60 to 0.85 and HR 0.79, 95% CI, 0.63 to 0.99, resp.). In particular, the hormone receptor positive subgroup had a benefit from response-guided therapy [18]. This fact could potentially explain the lack of correlation between pCR rate and survival, because, as previously mentioned, pCR rate is no good surrogate parameter in slowly proliferating tumors.

In *GeparQuinto*, the HER2 negative subgroup not responding to 4 cycles of epirubicin plus cyclophosphamide (EC) with or without bevacizumab was randomized to receive weekly paclitaxel plus everolimus or paclitaxel alone. The addition of everolimus did not change pCR rate (3.6 versus 5.6%, *P* = .48) but resulted in higher toxicity rates [19]. Before drawing definite conclusions, long-term results have to be awaited, because as indicated by *GeparTrio*, pCR may not be the appropriate endpoint.

Altogether, both trials showed that the pCR rates are low in patients not responding to the initial 2 to 4 cycles of chemotherapy and this could not be improved by changing the regimen. Nonetheless, response-guided therapy is a promising approach to optimize and individualize treatment for early breast cancer. An overview of the mentioned trials is given in Table 3.

## 7. Dose-Dense and Dose-Intensified Chemotherapy

Adjuvant as well as neoadjuvant trials tried to increase efficacy by shortening cycle intervals or by intensifying treatment dose. In the *GeparDuo* trial, dose-dense doxorubicin plus docetaxel was inferior to doxorubicin plus cyclophosphamide followed by docetaxel in respect to pCR rate [20]. Similarly, the *SWOG 0012* study, comparing a 15-week dose-dense regimen of weekly doxorubicin plus oral cyclophosphamide, did not show any difference in pCR rate, DFS or OS [21].

In contrast, the *AGO-1*, conducted by the GBG, showed a significant improvement in pCR rate (18 versus 10%, *P* = .008), DFS (HR 0.71, *P* = .011), and OS (HR 0.83, *P* = .041) when giving sequential epirubicin and paclitaxel in a dose-dense and dose-intensified manner compared with conventionally dosed concurrent epirubicin and paclitaxel. All patients received 3 cycles of CMF postoperatively [22]. With a very similar design, the *PREPARE* trial tried to increase response by applying CMF preoperatively instead of postoperatively. In fact, the pCR rate was better in the dose-dense and dose-intensified arm (19 versus 13%, *P* = .043), but without translating in a better DFS or OS (HR 1.14 and 1.26, resp.). The triple negative subgroup seemed to benefit most from intensively dosed chemotherapy even if not statistically significant (pCR: 45 versus 31%, *P* = .12) [23, 24]. This observation was confirmed in a pooled analysis of 7 trials of the GBG [25].

In general, dose-dense or dose-intensified treatment is associated with a higher incidence of grade 3/4 toxicities, even if febrile neutropenia can effectively be avoided by the use of G-CSF support. Notably, the incidence of secondary acute myeloid leukemia (AML) and myelodysplastic syndrome (MDS) is strongly correlated with dose intensity and could even be raised by the use of G-CSF [26]. Although the cumulative incidence is generally low (0.27–0.5% [26, 27]), such long-term effects might negatively influence survival after neoadjuvant treatment. This applies similarly to cardiotoxicity.

Therefore, and because of the modest impact on disease recurrence and OS in an unselected population, the dose-dense strategy cannot be recommended outside of clinical trials. Further studies are needed not only to define the optimal regimen but also to specify the patient population with the greatest benefit from dose-dense strategy (Table 4).

## 8. Targeting Her2

### 8.1. Trastuzumab

The first neoadjuvant phase-III trial investigating trastuzumab in combination with chemotherapy was prematurely terminated because of the impressive superiority of the experimental arm [28]. This success story was continued by the *NOAH* trial, which showed a significant improvement both in terms of pCR rate (43 versus 22%, *P* = .0007) and in 3-year event-free survival (71 versus 56%, *P* = .013) by the addition of trastuzumab to 3 cycles of doxorubicin plus paclitaxel, followed by 4 cycles of paclitaxel alone, and followed by 3 cycles of CMF [16]. A similar rate of pCR was achieved in a phase-II trial called *TECHNO*, where trastuzumab was added to 4 neoadjuvant cycles of paclitaxel following 4 cycles of EC [29]. Out of 1,509 participants treated in the GeparQuattro trial, 445 had HER2-positive tumors and received trastuzumab concomitantly to docetaxel or docetaxel plus capecitabine. The pCR rate in breast and axilla (ypT0/N0) differed significantly from HER2-negative tumors (32 versus 16%, *P* < .001) without influencing the rate of BCT [30].

### 8.2. Lapatinib

The subsequently conducted trials (*Neo-ALLTO*, *CherLob*, *NSABP-B41*, and *GeparQuinto*) compared the efficacy of the antibody trastuzumab with the one of the small molecule lapatinib [31–34]. Except *GeparQuinto*, none of them showed any difference in the pCR rate between both single agents. Lapatinib, however, was more toxic, mainly due to diarrhea (grade 3/4 in 12–36%). In the HER2-positive part of *GeparQuinto*, trastuzumab showed higher pCR rates than lapatinib (30 versus 23%, *P* = .04) [34]. *Neo-ALLTO*, *CherLob*, and *NSABP-B41* included each an arm where both compounds were combined. In all three trials the combination arm demonstrated higher efficacy than both comparative arms, although results in the *NSABP* trial were not statistically significant. Trastuzumab plus lapatinib in combination with chemotherapy led to pCR rates above 50%, without showing relevant cardiac safety issues [31–33].

This dual HER2 blockade might be the new standard in the neoadjuvant treatment of HER2-positive breast cancer, but until now, no survival data are available, and lapatinib is not approved for this indication. Similarly to the adjuvant *ALLTO* trial, the targeted therapy in *Neo-ALLTO* proceeds for a total of one year postoperatively. Thereby, long-term outcome shall be improved, particularly in case of non-pCR.

### 8.3. Pertuzumab

Pertuzumab is a monoclonal antibody blocking dimerization of HER2 with other members of the ERB-family like HER1, HER3, and HER4 as well as homodimerization. In the phase-II *NeoSphere* trial, 417 patients with HER2-positive breast cancer (≥2 cm) were randomized to 4 treatment arms each compromising 4 cycles: (A) docetaxel plus trastuzumab, (B) docetaxel plus trastuzumab plus pertuzumab, (C) trastuzumab plus pertuzumab without chemotherapy, and (D) docetaxel plus pertuzumab. The highest pCR rate, which was the primary endpoint, was attained in arm (B) with 46%, being significantly better than arm (A) and (D) with 29% and 24% (*P* = .0141 and *P* = .003), respectively. The chemotherapy-free arm showed an impressive pCR rate of 17% [35].

In *TRYPHAENA*, another phase-II trial, the combination of pertuzumab and trastuzumab was given in all three experimental arms: in arm (A) together with FEC followed by docetaxel, in arm (B) concomitantly to docetaxel following FEC and in arm (C) together with docetaxel and carboplatin. All three arms showed promising pCR rates of about 60% regardless of the chemotherapy chosen. Additionally, pertuzumab offers a favorable safety profile with neutropenia and drug hypersensitivity being the only grade 3/4 toxicities reported in the chemotherapy free-arm (0.9 and 1.9%, resp.). Even in combination with trastuzumab, no relevant changes in the left ventricular ejection fraction during neoadjuvant treatment were observed [36].

These promising phase-II data have now to be confirmed in larger phase-III trials, and long-term data have to be awaited. The results of the adjuvant trial *Aphinity* investigating the addition of pertuzumab to a trastuzumab-chemotherapy combination will also be of high interest. An overview of the most important neoadjuvant phase-III trials in HER2 positive cancer is given in Table 5.

## 9. Targeting VEGF

Two neoadjuvant trials with bevacizumab were published simultaneously in the New England Journal of Medicine: *GeparQuinto* (HER2 negative part) and *NSABP B-40* [10, 37]. Both phase-III trials showed a statistically significant benefit in terms of pCR rate by the addition of bevacizumab (18 versus 15%, *P* = .04 and 35 versus 28%, *P* = .02, resp.). Unfortunately, in the two studies, the subgroups with the greatest benefit from adding an antiangiogenic agent were different. In *GeparQuinto*, TNBC showed a more pronounced effect; in the *NSABP* trial, it was the hormone receptor-positive subgroup. Some potential explanations for this disparity are (1) that in GeparQuinto hormone receptor positive tumors were included only if they were also clinically node positive, (2) that the number of treatment cycles differed and (3) that in *NSABP B-40* a lower dose of docetaxel was given due to the combination with gemcitabine or capecitabine. It is worth mentioning that the *NSAPB* examines the postoperative continuation of bevacizumab for 10 cycles. However, a press release already announced that *BEATRICE*, investigating bevacizumab in the adjuvant setting, failed to reach its primary endpoint.

Neoadjuvant bevacizumab in combination with chemotherapy could become a new standard for Her2 negative breast cancer if the improvement of pCR can be translated in an improvement of long term outcome and if predictive markers can be found to reduce the number needed to treat.

## 10. Future Directions

A great number of well-known and new substances are in clinical testing in phase-II and phase-III trials. The goals of these trials are not only to improve pCR rates and survival but also to individualize therapy and to reduce toxicity. By combining chemotherapy with targeted therapy, primary or secondary resistance may be avoided. Dual, triple, or multiple simultaneous targeting will soon enter the clinic, probably allowing chemotherapy-free therapies for a certain subgroup of patients. To achieve this goal, the identification of predictive markers to determine patients with maximal benefit from a certain compound is of urgent need. This is particularly true for bevacizumab, where data about biomarkers from GeparQuinto, NSABP B-40, and BEATRICE are awaited.

### 10.1. HER2-Positive Breast Cancer

The fastest dynamic in research can be observed in HER2-positive breast cancer. 

Already, four drugs inhibiting the HER2 pathway are available (trastuzumab, lapatinib, pertuzumab, and trastuzumab emtansine). To overcome resistance to a HER2 blockade, several new substances, blocking HER2 itself or interacting molecules, are in clinical investigation. Ertumaxomab, for example, is a trifunctional, bispecific monoclonal antibody targeting HER2 and CD3. Neratinib, in contrast, is a tyrosine kinase inhibitor blocking HER1 and HER2 irreversibly.

Other approaches are tumor vaccines against HER2, a dual HER2 and insulin-like growth factor-1 receptor (IGF-1R) blockade, and inhibition of heat-shock protein 90 (HSP90).

### 10.2. Serial Biopsies and *Window-of-Opportunity* Trials

For a patient tailored therapy, biomarkers in tumor tissue or circulation, predicting treatment response or resistance, are needed. Due to the anatomic location of breast cancer, sequential biopsies during neoadjuvant treatment can be performed easily. Thereby, treatment-induced molecular changes can be monitored at an early time point to identify patients which respond. This principle has been shown for neoadjuvant endocrine therapy, where the extent of Ki-67 expression after only 2 weeks of treatment was significantly correlated with survival [38]. 

In addition to the classical neoadjuvant trial, a new design could be of greater interest: the so-called *window-of-opportunity* trial. In this case, a short course of targeted therapy is given prior to surgical resection or prior to standard therapy. The endpoint of such trials is not necessarily response rate but changes of biological markers, for example, for apoptosis or proliferation. A hypothesis-generating trial like this was done with metformin in operable breast cancer, where metformin was given twice daily for a median of 18 days prior to surgery. Ki-67 staining in invasive tumor tissue decreased significantly (from 37 to 34%, *P* = .016) and TUNEL staining increased (from 0.56 to 1.05, *P* = .004) [39]. 

*Window-of-opportunity *studies can be initiated to prove the expected mechanism of action, to identify tumor resistance and sensitivity, or to establish a “biologically effective” dose of the investigated targeted agent.

### 10.3. Postneoadjuvant Trials

Another field for new study designs will be the postneoadjuvant situation. Patients not achieving a pCR will be randomized postoperatively to a new therapy or to the established standard treatment, if available. Such a trial design is planned to compare trastuzumab emtansine (T-DM1) with classical trastuzumab as a treatment for patients with residual invasive disease following standard neoadjuvant therapy in HER2-positive cancer. The goal of post-neoadjuvant trials is to improve the poor prognosis of nonluminal breast cancer patients in case of residual disease.

### 10.4. New Registration Pathway

In May 2012, the FDA announced to reconsider the actual process of marketing approval for drugs in the neoadjuvant setting. Historically, new drugs for breast cancer had to be approved in the metastatic setting upfront, followed by adjuvant trials requiring many years of followup. As a result, the time from initiation of a phase III trial in the metastatic setting to approval for adjuvant use is often well more than a decade. Therefore, the FDA initiated a discussion to use pCR as an endpoint in high risk breast cancer to support approval under the accelerated approval regulations. To this end, the Collaborative Trials in Neoadjuvant Breast Cancer (CTNeoBC) working group, harboring investigators of different international study groups, has initiated a large meta-analysis to clarify the relationship between pCR and DFS/OS using primary source data from more than 12,000 patients enrolled in published randomized neoadjuvant trials. This potential new registration pathway would accelerate availability of new drugs and therefore help addressing an unmet need in these high risk populations.

## Figures and Tables

**Table 1:** Trials comparing the same chemotherapeutic regimen pre- and postoperatively.

Trial	Phase (*n*)	Tumors	NA versus adjuvant	Primary endpoint	Other outcomes	Ref.
IBBGS	III (272)	T2 > 3 cm or T3 N0-1	3 × EVM → 3 × ETV	BCT 63% (33% RT only, 30% S + RT) versus 0%	No difference in DFS or OS; 34% local recurrence with RT only	[40, 41]

Institut Curie S6	III (390)	T2-3, N0-1	4 × FAC	BCT 82 versus 77% (ns) (S only if no cCR after RT)	No difference in DFS and OS, short-term OS benefit (*P* = .02) for NA	[42, 43]

Royal Marsden	III (293)	T0–4, N0-1	4 × 2MT	BCT 89 versus 78% (*P* = .004)	No difference in DFS, OS, and local recurrence; pCR 7%	[44, 45]

NSABP B-18	III (1493)	T1–3, N0-1	4 × AC	5 y-OS: 80 versus 81% (ns); 5 y-DFS: 67 versus 67% (ns)	BCT 68 versus 60% (*P* = .001); LRR 13 versus 10% (*P* = .21); ORR 78%, pCR 13%; pCR associated with better 9 y-DFS (75 versus 58%) pCR associated with better 9 y-OS (85 versus 73%); trends in favor of NA for DFS and OS in women <50 y	[1, 46, 47]

EORTC 10902	III (698)	T1c–T4b	4 × FEC	4 y-OS 82 versus 84% (*P* = .38)	4 y-PFS 65 versus 70% (*P* = .27); LRR 5 versus 5% (ns); pCR 4%; downstaging to BCT in 23%	[2]

ABCSG-7	III (423)	T1–3, N0-1 HR− + high risk HR+	3 × CMF	RFS better with adjuvant therapy (HR 0.7; *P* = .02); no difference in OS (HR 0.8; *P* = .21)	cORR 56%, pCR 6%; LRR 13 versus 8% (*P* = .1)	[48]

Meta-analysis	IV (3946)	9 randomized trials	Same regimen	No difference in OS (RR 1.0); no difference in DFS (RR 0.99)	LRR higher for NA (RR 1.22; *P* = .015) especially if no S was done; pCR range 4–29%	[3]

EVM: epirubicin, vincristin, methotrexat; ETV: mitomycin, thiotepa, vindesine; FAC: 5-FU, doxorubicin, cyclophosphamide; 2MT: mitoxantrone, methotrexat, tamoxifen; AC: doxorubicin, cyclophosphamide; FEC: 5-FU, epirubicin, cyclophosphamide; CMF: cyclophosphamide, methotrexat, 5-FU.

**Table 2:** Randomized trials incorporating either concurrent or sequential taxane-based neoadjuvant therapy.

Trial	Phase (*n*)	Tumors	Treatment	Primary endpoint	Other outcomes	Ref.
Aberdeen trial	III (162)	≥3 cm	4 × CVAP → PR/CR: 4 × CVAP versus 4 × Doc; SD/PD: 4 × Doc	pCR 16 versus 34%; *P* = .04	cORR 66 versus 94%, *P* = .001; BCT 48 versus 67%; 5 y-OS (78 versus 93%, *P* = .04); 5 y-DFS (72 versus 90%, *P* = .04)	[49, 50]

NSABP B-27	III (2411)	T1c–3 N0, T1–3 N1; (median 9 cm)	4 × AC → S versus 4 × AC → 4 × Doc → S versus 4 × AC → S → 4 × Doc	DFS (arm 2 versus 1) HR 0.92 (*P* = .29); OS (*P* across all 3 arms = .76); RFI (arm 2 versus 1: HR 0.83, *P* = .04)	LRR (arm 2/3 versus 1) HR 0.67 (*P* = .02); BCT 62 versus 64% (ns), ORR 86 versus 91% (*P* < .001); pCR 9 versus 19% (*P* = .0001), pCR associated with better DFS (HR 0.49, *P* < .0001) and OS (HR 0.36, *P* < .0001)	[1, 51, 52]

ACCOG	III (363)	≥3 cm or T4d	6 × AC versus 6 × ADoc	pCR 24 versus 21% (*P* = .61); cORR 61 versus 70% (*P* = .06)	No difference in RFS (*P* = 17); no difference in OS (*P* = .57)	[53]

Diéras et al.	III (200)	T2-3 N0-1	4 × APac versus 4 × AC	pCR 16 versus 10% (*P* = NA)	cORR 89 versus 70%; BCT 58 versus 45%; DFS (18 MO: 87 versus 79%); pCR associated with better DFS (31 MO: 91 versus 70%)	[54]

Meta-analysis	IV (2455)	7 randomized trials	Anthracycline-based therapy ± taxane	pCR better with sequential (RR 1.73, *P* = .013), but not with concomitant taxanes (RR 1.04, *P* = .77); BCT higher with taxanes (RR 1.11, *P* = .012)	No difference in DFS (RR 0.91, *P* = .12)	[6]

CVAP: cyclophosphamide, vincristin, doxorubicin, prednisone; Doc: doxetaxel; AC: docorubicin, cyclophosphamide; Pac: paclitaxel.

**Table 3:** Trials investigating response-adapted therapy.

Trial	Phase (*n*)	Tumors	Treatment	Primary endpoint	Other outcomes	Ref.
GeparTrio pilot	II (285)	≥2 cm	2 × TAC → PR/CR: 4 × TAC; SD: 4 × TAC versus 4 × NX	pCR 23 versus 7 versus 3%	BCT 72%; pCR 57% <40 y with TNBC or G3)	[55]

GeparTrio	III (2012)	T2–4 N0–3 (except T2 + ER/PR pos. + cN0 + G1/2 + >35 y)	2 × TAC → PR/CR: 4 versus 6 × TAC; SD: 4 × TAC versus 4 × NX	pCR 6 versus 8 × TAC: 21 versus 24% (*P* = .27); pCR TAC versus NX: 5 versus 6% (*P* = .73)	cORR 6 versus 8 × TAC: 75 versus 74% (ns); cORR TAC versus NX: both 51% (*P* for noninferiority = .008); BET 68 versus 59% (*P* = .001); NX less toxic; PFS 6 versus 8 × TAC versus TAC-NX: HR 0.71 (*P* = .001); OS 6 versus 8 × TAC versus TAC-NX: HR 0.79 (*P* = .048) Benefit of response-guided therapy derives from HR+ tumors (no benefit for HER2+/HR− and TNBC)	[18, 56–58]

GeparQuinto (HER2 negative)	III (403)	cT3/4; cT2 if HR− or cN+; cT1 if HR− or SLN+	4 × EC ± Bev → SD: 12 × Pac ± Rad001	pCR 4 versus 6% (*P* = .48)	cORR 52 versus 62%; toxicity higher in the everolimus group	[19]

TAC: docetaxel, doxorubicin, cyclophosphamide; Pac: paclitaxel; NX: vinorelbine, capecitabine; EC: epirubicin, cyclophosphamide; Bev: bevacizumab; Rad001: everolimus.

**Table 4:** Trials investigating dose-dense and dose-intensified neoadjuvant chemotherapy.

Trial	Phase (*n*)	Tumors	Treatment	Primary endpoint	Other outcomes	Ref.
GeparDuo	III (904)	T2-3 N0–2,	4 × DD ADoc, q14 + G-CSF versus 4 × AC → 4 × Doc, q21	pCR 7 versus 14% (*P* < .001) (closed early because of pCR difference)	BCT 58 versus 63% (*P* = .05); cORR 69 versus 79% (*P* < .001); 5 y-EFS 65 versus 66% (*P* = .66); 5 y-OS 81 versus 85% (*P* = .24); trend for an improved DFS and OS for pts achieving a pCR (recurrence/death, 22/12% versus 29/17%, *P* = .37/.32)	[20, 59, 60]

AGO-1	III (668)	≥3 cm or cT4d	3 × IDD E → 3 × Pac, q14 versus 4 × E + 4 × Pac, q21 + 3 × CMF after S for all	pCR 18 versus 10% (*P* = .008)	BCS 55 versus 50% (*P* = .26); 5 y-DFS 70 versus 59% (HR 0.71, *P* = .011); 5 y-OS 83 versus 77% (HR 0.83, *P* = .041); no benefit for inflammatory BC; more nonhematologic toxicities, anemia, and thrombocytopenia, but similar neutropenia and infection rates	[22]

PREPARE	III (733)	≥2 cm or cT4d	3 × IDD E → 3 × Pac + G-CSF → CMF, q14 versus 4 × EC → 4 × Pac, q21 ± DA	3 y-DFS 79 versus 76% (*P* = .37)	3 y-OS 92 versus 88% (*P* = .24); pCR 19 versus 13% (*P* = .043); pCR associated with better DFS (*P* = .001); 3 y-DFS DA + versus −: 74 versus 80% (*P* = .061)	[23, 24]

SWOG 0012	III (372)	IIB-IIIB	5 × AC → 12 × Pac weekly versus 15 × A weekly + C daily p.o. + G-CSF	pCR 21 versus 24% (*P* = .45)	No difference in DFS (HR 1.03, *P* = .87); no difference in OS (HR 1.19, *P* = .37)	[61]

MDACC	III (202)	IIA–IV	FAC q21 versus DI FAC + G-CSF q18	pCR 9 versus 13% (*P* = .35)	cORR 77 versus 92% (*P* = .003); 5 y-OS 66 versus 67% (*P* = .61); 5 y-DFS 56 versus 67% (*P* = .12)	[62]

DD: dose dense; IDD: intensified dose dense; DI: dose intensified; ADoc: doxorubicin, docetaxel; AC: doxorubicin, cyclophosphamide; Doc: docetaxel; Pac: paclitaxel; CMF: cyclophosphamide, methotrexat, 5-FU; DA: darbepoetin alpha; C: cyclophosphamide; FAC: 5FU, doxorubicin, cyclophosphamide; A: doxorubicin.

**Table 5:** The most important neoadjuvant phase-III trials in HER2-positive cancer.

Trial	Phase (*n*)	Tumors	Treatment	Primary endpoint	Other outcomes	Ref.
Buzdar et al.	III (42/164)	HER2+, II-IIIA	4 × Pac → 4 × FEC ± H (24 × weekly)	pCR 67 versus 25% (*P* = .02); (closed early because of pCR difference)	cORR 96 versus 84% (*P* = na); no clinical cardiac failure; >10% decrease in LVE 7 versus 5 patients	[28]

NOAH	III (99 Her2+/343)	T3 N1 or T4 or N2-3	3 × APac → 4 × Pac → 3 × CMF ± H	3 y-EFS 71 versus 56% (*P* = .013)	bpCR 43 versus 22% (*P* = .0007); tpCR 38 versus 19% (*P* = .001); 3 y-OS 87 versus 79% (*P* = .114); despite concurrent administration with doxorubicin CHF only 2%	[16]

GeparQuattro	III (445 HER2+/1509)	T1c–4d N0–3 (N0 only if HR−)	4 × EC → 4 × Doc + H (HER2+) ± X (combination or sequence)	tpCR 32 versus 16% (*P* < .001)	BCT 63 versus 65% (ns); pCR Doc versus Doc + X versus Doc→ X 22 versus 20 versus 22% (ns); BCT 70 versus 68 versus 65% (ns)	[12, 30]

TECHNO	II (217)	HER2+, ≥2 cm or cT4d	4 × EC → 4 × Pac + H	pCR 39%	BCT 65%; CHF 3.7% 3 y-DFS 78% 3 y-DFS pCR versus non: 96 versus 86% (*P* = .003)	[29]

GeparQuinto (Her2 positive)	III (620)	cT3/4; cT2 if HR− or cN+; cT1 if HR− or SLN+	4 × EC → 4 × Doc + H versus L	pCR 30 versus 23% (*P* = .04)	cORR 90% both (ns) diarrhea ≥ gr 3: 3 versus 12% (*P* < .0001) CHF 1 versus 7 (ns)	[34]

CHER-LOB	II (121)	HER2+, II-IIIA	12 × Pac → 4 × FEC + H versus L versus L + H	pCR 25 versus 26 versus 47% (L/H versus L + H: *P*_exploratory_ = .019)	BCT 67 versus 58 versus 70%; diarrhea ≥ gr 3: 3 versus 36 versus 35%	[32]

NEO-ALTTO	III (455)	HER2+, ≥2 cm	6 × H (w) versus L versus L + H (w) → combination with 12 × Pac (w) → S → FEC + same schedule for 1 y	pCR 30 versus 25 versus 51% (H versus L + H: *P* = .0001; H versus L: *P* = .34)	cORR after 6 weeks 30 versus 53 versus 67% (both *P* < .0001); cORR at surgery 71 versus 74 (*P* = .49) versus 80% (*P* = .049); diarrhea ≥ gr 3: 2 versus 23 versus 21%	[63]

NSABP B-41	III (522)	HER2+, ≥2 cm	4 × AC → 4 × Doc + H (w) versus L versus L + H (w) (+H for 1 y adjuvant for all)	pCR 53 versus 53 versus 62% (H versus L + H: *P* = .075; H versus L: *P* = .9)	diarrhea ≥ gr 3: 2 versus 20 versus 27% (*P* < .001) CHF ≥ gr 3: 4 versus 4 versus 2% (*P* = 0.49)	[33]

NeoSphere	II (417)	HER2+, ≥2 cm or cT4d	(A) 4 × Doc + H versus (B) 4 × Doc + H + P versus (C) H + P versus (D) 4 × Doc + P	pCR 29 versus 46 versus 17 versus 24% (B versus A: *P* = .0141; C versus A: *P* = .019; B versus D: *P* = .003)	cORR 81 versus 88 versus 66 versus 74%	[35]

TRYPHAENA	II (225)	HER2+, II-III	3 × FEC + H + P → 3 × Doc + H + P versus 3 × FEC → 3 × Doc + H + P versus 6 × Doc + Carbo + H + P	Cardiac safety: symptomatic LVSD 0 versus 2.7 versus 0%	pCR 62 versus 57 versus 66%; cORR 92 versus 95 versus 90%	[36]

Pac: paclitaxel; FEC: 5FU, epirubicin, cyclophosphamide; H: trastuzumab; APac: doxorubicin, paclitaxel; X: capecitabine; L: lapatinib; w: weekly; Doc: docetaxel; Carbo: carboplatin; na: not available; bpCR: pathological complete response in breast tissue; tpCR: total pathological complete response (inbreast and axillary nodes); LVSD: left ventricular end-systolic dimension.

## Abbreviations

2MT: Mitoxantrone, metotrexat, tamoxifen
A: Doxorubicin
AC: Doxorubicin, cyclophosphamide
ADoc: Doxorubicin, docetaxel
APac: Doxorubicin, paclitaxel
Bev: Bevacizumab
BCT: Breast conserving therapy
bpCR: Pathological complete response in breast tissue
C: Cyclophosphamide
Carbo: Carboplatin
cCR: Clinical complete response
CHF: Chronic heart failure
CMF: Cyclophosphamide, metotrexat, 5-FU
cORR: Clinical overall response rate
cPR: Clinical partial response
CVAP: Cyclophosphamide, vincristine, doxorubicin, prednisone
DA: Darbepoetin alpha
DD: Dose dense
DI: Dose intensified
DFS: Disease-free survival
Doc: Docetaxel
EC: Epirubicin, cyclophosphamide
ETV: Mitomycin, thiotepa, vindesin
EVM: Epirubicin, vincristine, metotrexat
FEC: 5-FU, epirubicin, cyclophosphamide
FAC: 5FU, doxorubicin, cyclophosphamide
H: Trastuzumab
IDD: Intensified dose-dense
L: Lapatinib
LRR: Local recurrence rate
LVSD: Left ventricular end-systolic dimension
MO: Months
na: Not available
NA: Neoadjuvant
NX: Vinorelbine, capecitabine
OS: Overall survival
Pac: Paclitaxel
pCR: Pathologic complete response
Rad001: Everolimus
RCB: Residual cancer burden
RFS: Recurrence-free survival
RT: Radiotherapy
S: Surgery
TAC: Docetaxel, doxorubicin, cyclophosphamide
tpCR: Total pathological complete response (inbreast and axillary nodes)
N: Vinorelbine
X: Capecitabine.
